# Ethyl 7-(2-chloro­phen­yl)-5-trifluoro­meth­yl-4,7-dihydro-1,2,4-triazolo[1,5-*a*]pyrimidine-6-carboxyl­ate

**DOI:** 10.1107/S1600536810037712

**Published:** 2010-09-25

**Authors:** Jie Mou, Chen-Xia Yu, Chang-Sheng Yao

**Affiliations:** a School of Pharmacy, Xuzhou Medical College, Xuzhou 221004, People’s Republic of China; b School of Chemistry and Chemical Engineering, Xuzhou Normal University, Xuzhou 221116, People’s Republic of China; c Key Laboratory of Biotechnology for Medicinal Plants, Xuzhou Normal University, Xuzhou 221116, People’s Republic of China

## Abstract

In the title compound, C_15_H_12_ClF_3_N_4_O_2_, the dihydro­pyrimidine ring exhibits an envelope conformation. The dihedral angle between the mean planes of the dihydro­pyrimidine and phenyl rings is 83.94 (6)°. The OCH_2_CH_3_ group is disordered over two sites with occupancies of 0.155 (3) and 0.845 (3). The crystal packing is stabilized by inter­molecular N—H⋯N hydrogen bonds.

## Related literature

For the anti­cancer activity, inhibition of the MDM2-p53 protein–protein inter­action and the anti­tuberculosis and dehydrogenase inhibitory activity of [1,2,4]triazolo [1,5-*a*]pyrimidine derivatives, see: Zhang *et al.* (2007 ▶); Allen *et al.* (2009 ▶); Pereyaslavskaya *et al.* (2008 ▶); Gujjar *et al.* (2009 ▶). For the bioactivity of trifluoro­methyl­ated mol­ecules, see: Kirk, (2006 ▶). For the preparation of trifluoro­methyl­ated [1,2,4]triazolo[1,5-*a*]pyrimidine derivatives, see Pryadeina *et al.* (2004 ▶). For puckering parameters, see: Cremer & Pople (1975 ▶).
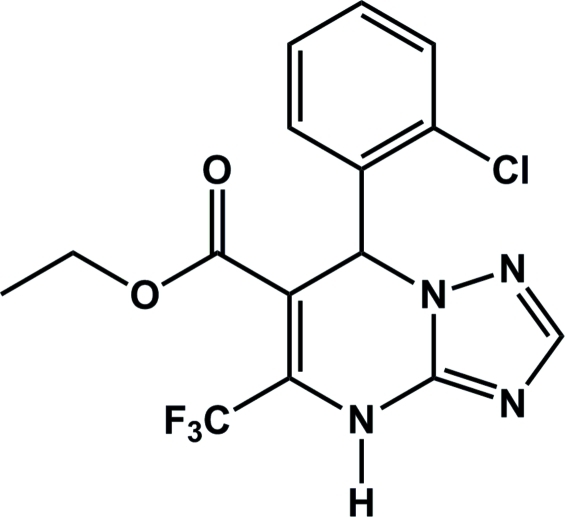


## Experimental

### 

#### Crystal data

C_15_H_12_ClF_3_N_4_O_2_
*M* *_r_* = 372.74
Monoclinic, 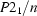

*a* = 9.8927 (12) Å
*b* = 6.8055 (6) Å
*c* = 24.403 (3) Å
β = 99.237 (9)°
*V* = 1621.6 (3) Å^3^
*Z* = 4
Mo *K*α radiation
μ = 0.29 mm^−1^
*T* = 113 K
0.26 × 0.22 × 0.20 mm

#### Data collection

Rigaku Saturn diffractometer
Absorption correction: multi-scan (*CrystalClear*;Rigaku/MSC, 2002 ▶) *T*_min_ = 0.930, *T*_max_ = 0.945
14364 measured reflections
3835 independent reflections
3058 reflections with *I* > 2σ(*I*)
*R*_int_ = 0.043

#### Refinement

*R*[*F* ^2^ > 2σ(*F* ^2^)] = 0.047
*wR*(*F* ^2^) = 0.121
*S* = 1.08
3835 reflections
242 parameters
6 restraints
H atoms treated by a mixture of independent and constrained refinement
Δρ_max_ = 0.27 e Å^−3^
Δρ_min_ = −0.40 e Å^−3^

### 

Data collection: *CrystalClear* (Rigaku/MSC, 2002 ▶); cell refinement: *CrystalClear*; data reduction: *CrystalClear*; program(s) used to solve structure: *SHELXS97* (Sheldrick, 2008 ▶); program(s) used to refine structure: *SHELXL97* (Sheldrick, 2008 ▶); molecular graphics: *SHELXTL* (Sheldrick, 2008 ▶); software used to prepare material for publication: *SHELXTL*.

## Supplementary Material

Crystal structure: contains datablocks I, global. DOI: 10.1107/S1600536810037712/hg2715sup1.cif

Structure factors: contains datablocks I. DOI: 10.1107/S1600536810037712/hg2715Isup2.hkl

Additional supplementary materials:  crystallographic information; 3D view; checkCIF report

## Figures and Tables

**Table 1:** Hydrogen-bond geometry (Å, °)

*D*—H⋯*A*	*D*—H	H⋯*A*	*D*⋯*A*	*D*—H⋯*A*
N1—H1⋯N4^i^	0.90 (2)	1.96 (2)	2.843 (2)	166.3 (19)

Symmetry code: (i) 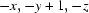.

**Fig. 1.**
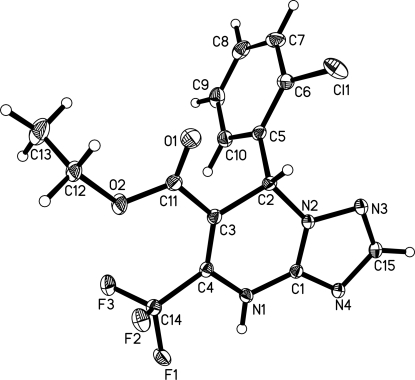
The molecular structure of the title compound, showing 30% probability displacement ellipsoids and the atom-numbering scheme. The minor part of the disordered moieties were omitted for clarity.

**Fig. 2.**
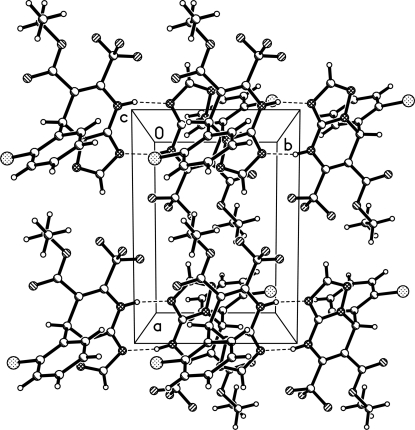
A packing diagram of the title compound. Intermolecular hydrogen bonds are shown as dashed lines. The minor part of the disordered moieties were omitted for clarity.

## supplementary crystallographic information

### Comment

1,2,4-Triazolo[1,5-a]pyrimidine derivatives are known because of their wide range of biological activities. For example, some of the trizaolopyrimidines can be used as anticancer agents (Zhang *et al.*, 2007), inhibitor of the MDM2-p53 protein-protein interaction (Allen *et al.*, 2009), antituberculosis agents (Pereyaslavskaya *et al.*, 2008) and dehydrogenase inhibitors (Gujjar *et al.*, 2009). Therefore, the preparation or structural modification of these nitrogen-containing heterocyclic scaffolds is of considerable interest for both organic and medicinal chemistry. The introduction of a trifluoromethyl group into organic molecules often changes their physical, chemical, and physiological properties (Kirk, 2006). During the synthesis of trifluoromethylated 1,2,4-Triazolo[1,5-*a*]pyrimidine derivatives, the title compound (I) was isolated and its structure was determined by X-ray analysis. The results are presented here.

In the title molecule (Fig. 1), the 1,2,4-triazole ring adopts a planar conformation.  Cremer & Pople puckering analysis (Cremer &Pople, 1975) can not be performed, for its weighted average absolute torsion angle is 0.7°, less than 5.0°. The dihydropyrimidine ring system is in an envelope conformation, for Cremer & Pople puckering analysis shows θ(2) and φ(2) are 0.094 (2)Å and 346.4 (10)°, respectively. Its puckering amplitude (Q) is 0.099 (2)Å. Besides, the distance between atom C2 and the mean N2/C1/N1/C4/C3 plane (r.m.s. deviation 0.016 Å) is 0.136 (2) Å, which also confirms the conformation of the dihydropyrimidine ring. The dihedral angle between the aforementioned weighted plane and phenyl ring is 83.94 (6)°, which shows the two units are nearly perpendicular.

The crystal packing is stabilized by intermolecular  N—H···N hydrogen bonds (Table 1, Fig.2).

### Experimental

The title compound was synthesized according the procedure reported by Pryadeina *et al.* (2004). A mixture of 0.01 mol of ethyl 4,4,4-trifluoro-3-oxobutanoate, 0.01 mol of 2-chlorobenzaldehyde and 0.01 mol of 1H-1,2,4-triazol-5-amine in 20 mL of ethanol containing a catalytic amount of hydrochloric acid was heated for 12 h under reflux. Then the solvent was removed under reduced pressure. The residue was added to a solution of p-toluenesulfonic acid, 0.05 g, in 100 mL of benzene, and the mixture was heated for 8 h with simultaneous removal of water as azeotrope with benzene. The solution was filtered while hot, the filtrate was evaporated, and the precipitate was recrystallized from ethanol. Cooling the ethanol solution slowly gave single crystals suitable for X-ray diffraction.

### Refinement

The H atoms bound to N atoms were located in a difference map and were refined freely [refined N–H length, 0.90 (2)Å]. All other H atoms were placed in calculated positions, with C–H = 0.95, 0.98, 0.99 or 1.00 Å, and included in the final cycles of refinement using a riding model, with U_iso_(H) = 1.2U_eq_(parent atom). The OCH_2_CH_3_ group is disordered over two sites with occupancies of 0.155 (3) and 0.845 (3). The atom pairs of C12 and C12', C13 and C13', and O2 and O2' are constrained to have the same anisotropic displacement parameters. The bond lengths of ethyl group of C12–C13 and C12'–C13' is restrained to 1.54Å with esd of 0.01Å. The distance between O2 and C12, O2' and C12' is restrained to 1.42Å with esd of 0.01Å. The atoms of O2 and O2' are restrained to be at the distance of 1.38Å from the atom of C11 with esd of 0.01Å.

### Crystal data

**Table:**

C_15_H_12_ClF_3_N_4_O_2_	*F*(000) = 760
*M**_r_* = 372.74	*D*_x_ = 1.527 Mg m^−^^3^
Monoclinic, *P*2_1_/*n*	Mo *K*α radiation, λ = 0.71070 Å
Hall symbol: -P 2yn	Cell parameters from 3832 reflections
*a* = 9.8927 (12) Å	θ = 2.4–27.9°
*b* = 6.8055 (6) Å	µ = 0.28 mm^−^^1^
*c* = 24.403 (3) Å	*T* = 113 K
β = 99.237 (9)°	Block, colorless
*V* = 1621.6 (3) Å^3^	0.26 × 0.22 × 0.20 mm
*Z* = 4	

### Data collection

**Table:**

Rigaku Saturn diffractometer	3835 independent reflections
Radiation source: rotating anode	3058 reflections with *I* > 2σ(*I*)
confocal	*R*_int_ = 0.043
Detector resolution: 7.31 pixels mm^-1^	θ_max_ = 27.9°, θ_min_ = 2.4°
ω scans	*h* = −13→13
Absorption correction: multi-scan *CrystalClear*	*k* = −8→8
*T*_min_ = 0.930, *T*_max_ = 0.945	*l* = −32→32
14364 measured reflections	

### Refinement

**Table:**

Refinement on *F*^2^	Primary atom site location: structure-invariant direct methods
Least-squares matrix: full	Secondary atom site location: difference Fourier map
*R*[*F*^2^ > 2σ(*F*^2^)] = 0.047	Hydrogen site location: inferred from neighbouring sites
*wR*(*F*^2^) = 0.121	H atoms treated by a mixture of independent and constrained refinement
*S* = 1.08	*w* = 1/[σ^2^(*F*_o_^2^) + (0.0596*P*)^2^ + 0.2191*P*] where *P* = (*F*_o_^2^ + 2*F*_c_^2^)/3
3835 reflections	(Δ/σ)_max_ = 0.001
242 parameters	Δρ_max_ = 0.27 e Å^−^^3^
6 restraints	Δρ_min_ = −0.40 e Å^−^^3^

### Special details

**Table:**

Geometry. All esds (except the esd in the dihedral angle between two l.s. planes) are estimated using the full covariance matrix. The cell esds are taken into account individually in the estimation of esds in distances, angles and torsion angles; correlations between esds in cell parameters are only used when they are defined by crystal symmetry. An approximate (isotropic) treatment of cell esds is used for estimating esds involving l.s. planes.
Refinement. Refinement of F^2^ against ALL reflections. The weighted R-factor wR and goodness of fit S are based on F^2^, conventional R-factors R are based on F, with F set to zero for negative F^2^. The threshold expression of F^2^ > 2sigma(F^2^) is used only for calculating R-factors(gt) etc. and is not relevant to the choice of reflections for refinement. R-factors based on F^2^ are statistically about twice as large as those based on F, and R- factors based on ALL data will be even larger.

### Fractional atomic coordinates and isotropic or equivalent isotropic displacement parameters (Å^2^)

**Table:**

	*x*	*y*	*z*	*U*_iso_*/*U*_eq_	Occ. (<1)
Cl1	−0.02142 (6)	1.41310 (7)	0.14247 (2)	0.04311 (18)	
F1	0.35904 (10)	0.54406 (16)	0.01321 (5)	0.0375 (3)	
F2	0.44949 (11)	0.82808 (16)	0.01419 (5)	0.0334 (3)	
F3	0.47647 (11)	0.65227 (16)	0.08811 (5)	0.0329 (3)	
O1	0.33660 (14)	1.2697 (2)	0.11875 (6)	0.0369 (3)	
N1	0.14288 (14)	0.6813 (2)	0.03869 (6)	0.0211 (3)	
N2	0.00227 (14)	0.9257 (2)	0.06666 (6)	0.0213 (3)	
N3	−0.13449 (15)	0.9673 (2)	0.06434 (6)	0.0261 (3)	
N4	−0.10446 (14)	0.6764 (2)	0.02200 (6)	0.0228 (3)	
C1	0.01658 (17)	0.7543 (2)	0.04212 (7)	0.0198 (3)	
C2	0.11062 (17)	1.0474 (2)	0.09691 (7)	0.0205 (3)	
H2	0.1003	1.1842	0.0819	0.025*	
C3	0.24727 (17)	0.9649 (2)	0.08603 (7)	0.0215 (4)	
C4	0.25580 (16)	0.7931 (2)	0.05905 (7)	0.0199 (3)	
C5	0.09913 (17)	1.0529 (2)	0.15815 (7)	0.0217 (4)	
C6	0.04123 (19)	1.2105 (3)	0.18215 (8)	0.0274 (4)	
C7	0.0304 (2)	1.2109 (3)	0.23816 (8)	0.0356 (5)	
H7	−0.0083	1.3206	0.2541	0.043*	
C8	0.0765 (2)	1.0501 (3)	0.27056 (8)	0.0381 (5)	
H8	0.0690	1.0490	0.3089	0.046*	
C9	0.1329 (2)	0.8925 (3)	0.24762 (8)	0.0355 (5)	
H9	0.1644	0.7823	0.2700	0.043*	
C10	0.14409 (19)	0.8941 (3)	0.19169 (8)	0.0284 (4)	
H10	0.1833	0.7842	0.1761	0.034*	
C11	0.36016 (18)	1.1040 (3)	0.10611 (7)	0.0249 (4)	
O2	0.48511 (16)	1.0381 (3)	0.10610 (10)	0.0390 (6)	0.845 (3)
C12	0.5990 (2)	1.1697 (4)	0.12467 (11)	0.0315 (6)	0.845 (3)
H12A	0.5678	1.3077	0.1199	0.038*	0.845 (3)
H12B	0.6721	1.1489	0.1019	0.038*	0.845 (3)
C13	0.6540 (3)	1.1316 (5)	0.18435 (12)	0.0518 (8)	0.845 (3)
H13A	0.5832	1.1607	0.2070	0.078*	0.845 (3)
H13B	0.7338	1.2157	0.1960	0.078*	0.845 (3)
H13C	0.6812	0.9934	0.1892	0.078*	0.845 (3)
O2'	0.4728 (9)	0.9900 (15)	0.1298 (5)	0.0390 (6)	0.155 (3)
C12'	0.5996 (11)	1.0954 (19)	0.1454 (7)	0.0315 (6)	0.155 (3)
H12C	0.5860	1.2056	0.1704	0.038*	0.155 (3)
H12D	0.6309	1.1499	0.1120	0.038*	0.155 (3)
C13'	0.7072 (14)	0.952 (2)	0.1752 (7)	0.0518 (8)	0.155 (3)
H13D	0.6847	0.9190	0.2117	0.078*	0.155 (3)
H13E	0.7979	1.0133	0.1796	0.078*	0.155 (3)
H13F	0.7076	0.8317	0.1530	0.078*	0.155 (3)
C14	0.38686 (17)	0.7050 (2)	0.04419 (7)	0.0225 (4)	
C15	−0.19178 (18)	0.8142 (3)	0.03716 (7)	0.0255 (4)	
H15	−0.2884	0.8001	0.0285	0.031*	
H1	0.146 (2)	0.567 (3)	0.0203 (9)	0.039 (6)*	

### Atomic displacement parameters (Å^2^)

**Table:**

	*U*^11^	*U*^22^	*U*^33^	*U*^12^	*U*^13^	*U*^23^
Cl1	0.0546 (4)	0.0261 (3)	0.0533 (4)	0.0094 (2)	0.0228 (3)	0.0025 (2)
F1	0.0217 (6)	0.0361 (6)	0.0564 (8)	−0.0049 (5)	0.0118 (5)	−0.0233 (5)
F2	0.0250 (6)	0.0366 (6)	0.0418 (7)	−0.0032 (4)	0.0153 (5)	0.0102 (5)
F3	0.0249 (6)	0.0370 (6)	0.0359 (6)	0.0063 (5)	0.0023 (5)	0.0070 (5)
O1	0.0310 (8)	0.0290 (7)	0.0506 (9)	−0.0071 (6)	0.0063 (6)	−0.0098 (6)
N1	0.0169 (7)	0.0207 (7)	0.0259 (8)	−0.0011 (5)	0.0038 (6)	−0.0039 (6)
N2	0.0150 (7)	0.0256 (7)	0.0231 (7)	0.0003 (5)	0.0022 (6)	−0.0043 (6)
N3	0.0169 (7)	0.0317 (8)	0.0293 (8)	0.0022 (6)	0.0026 (6)	−0.0046 (6)
N4	0.0172 (7)	0.0261 (8)	0.0245 (8)	−0.0008 (6)	0.0017 (6)	−0.0028 (6)
C1	0.0175 (8)	0.0224 (8)	0.0191 (8)	−0.0011 (6)	0.0022 (6)	−0.0002 (6)
C2	0.0194 (8)	0.0210 (8)	0.0208 (8)	−0.0021 (6)	0.0022 (6)	−0.0027 (6)
C3	0.0186 (8)	0.0250 (8)	0.0207 (8)	−0.0015 (7)	0.0030 (7)	−0.0007 (6)
C4	0.0168 (8)	0.0235 (8)	0.0194 (8)	−0.0023 (6)	0.0030 (6)	0.0023 (6)
C5	0.0177 (8)	0.0269 (9)	0.0205 (8)	−0.0036 (7)	0.0027 (7)	−0.0028 (7)
C6	0.0251 (9)	0.0283 (9)	0.0295 (10)	−0.0030 (7)	0.0069 (7)	−0.0045 (7)
C7	0.0325 (11)	0.0440 (12)	0.0329 (11)	−0.0062 (9)	0.0134 (8)	−0.0141 (9)
C8	0.0311 (11)	0.0631 (14)	0.0207 (9)	−0.0098 (10)	0.0057 (8)	−0.0034 (9)
C9	0.0281 (10)	0.0525 (12)	0.0260 (10)	0.0015 (9)	0.0043 (8)	0.0104 (9)
C10	0.0249 (9)	0.0341 (10)	0.0263 (9)	0.0022 (8)	0.0046 (7)	0.0019 (8)
C11	0.0215 (9)	0.0318 (10)	0.0217 (9)	−0.0031 (7)	0.0051 (7)	−0.0037 (7)
O2	0.0174 (8)	0.0325 (10)	0.0652 (16)	−0.0059 (7)	0.0001 (8)	−0.0180 (10)
C12	0.0184 (10)	0.0282 (14)	0.0467 (16)	−0.0069 (9)	0.0016 (10)	−0.0065 (11)
C13	0.0329 (15)	0.070 (2)	0.0506 (17)	−0.0184 (13)	0.0010 (12)	−0.0013 (15)
O2'	0.0174 (8)	0.0325 (10)	0.0652 (16)	−0.0059 (7)	0.0001 (8)	−0.0180 (10)
C12'	0.0184 (10)	0.0282 (14)	0.0467 (16)	−0.0069 (9)	0.0016 (10)	−0.0065 (11)
C13'	0.0329 (15)	0.070 (2)	0.0506 (17)	−0.0184 (13)	0.0010 (12)	−0.0013 (15)
C14	0.0197 (8)	0.0227 (8)	0.0258 (9)	−0.0040 (6)	0.0059 (7)	−0.0004 (7)
C15	0.0165 (8)	0.0313 (9)	0.0282 (9)	0.0003 (7)	0.0020 (7)	−0.0029 (7)

### Geometric parameters (Å, °)

**Table:**

Cl1—C6	1.7407 (19)	C7—H7	0.9500
F1—C14	1.3339 (19)	C8—C9	1.369 (3)
F2—C14	1.3293 (19)	C8—H8	0.9500
F3—C14	1.326 (2)	C9—C10	1.387 (3)
O1—C11	1.202 (2)	C9—H9	0.9500
N1—C1	1.359 (2)	C10—H10	0.9500
N1—C4	1.377 (2)	C11—O2	1.315 (2)
N1—H1	0.90 (2)	C11—O2'	1.404 (8)
N2—C1	1.329 (2)	O2—C12	1.453 (3)
N2—N3	1.3744 (19)	C12—C13	1.493 (4)
N2—C2	1.458 (2)	C12—H12A	0.9900
N3—C15	1.314 (2)	C12—H12B	0.9900
N4—C1	1.329 (2)	C13—H13A	0.9800
N4—C15	1.366 (2)	C13—H13B	0.9800
C2—C5	1.517 (2)	C13—H13C	0.9800
C2—C3	1.526 (2)	O2'—C12'	1.442 (9)
C2—H2	1.0000	C12'—C13'	1.539 (9)
C3—C4	1.351 (2)	C12'—H12C	0.9900
C3—C11	1.486 (2)	C12'—H12D	0.9900
C4—C14	1.524 (2)	C13'—H13D	0.9800
C5—C10	1.385 (2)	C13'—H13E	0.9800
C5—C6	1.389 (2)	C13'—H13F	0.9800
C6—C7	1.388 (3)	C15—H15	0.9500
C7—C8	1.384 (3)		
			
C1—N1—C4	118.45 (14)	C5—C10—C9	121.27 (18)
C1—N1—H1	116.7 (14)	C5—C10—H10	119.4
C4—N1—H1	124.6 (14)	C9—C10—H10	119.4
C1—N2—N3	109.72 (13)	O1—C11—O2	122.77 (17)
C1—N2—C2	127.16 (14)	O1—C11—O2'	125.9 (5)
N3—N2—C2	122.86 (13)	O2—C11—O2'	29.2 (5)
C15—N3—N2	101.51 (14)	O1—C11—C3	121.07 (16)
C1—N4—C15	101.41 (14)	O2—C11—C3	116.05 (16)
N4—C1—N2	111.19 (15)	O2'—C11—C3	106.8 (4)
N4—C1—N1	127.88 (15)	C11—O2—C12	118.11 (18)
N2—C1—N1	120.91 (15)	O2—C12—C13	109.9 (2)
N2—C2—C5	110.32 (13)	O2—C12—H12A	109.7
N2—C2—C3	107.61 (13)	C13—C12—H12A	109.7
C5—C2—C3	112.88 (14)	O2—C12—H12B	109.7
N2—C2—H2	108.6	C13—C12—H12B	109.7
C5—C2—H2	108.6	H12A—C12—H12B	108.2
C3—C2—H2	108.6	C11—O2'—C12'	115.7 (8)
C4—C3—C11	127.56 (16)	O2'—C12'—C13'	108.4 (10)
C4—C3—C2	121.99 (15)	O2'—C12'—H12C	110.0
C11—C3—C2	110.36 (14)	C13'—C12'—H12C	110.0
C3—C4—N1	122.93 (16)	O2'—C12'—H12D	110.0
C3—C4—C14	125.36 (15)	C13'—C12'—H12D	110.0
N1—C4—C14	111.62 (14)	H12C—C12'—H12D	108.4
C10—C5—C6	117.92 (16)	C12'—C13'—H13D	109.5
C10—C5—C2	119.70 (15)	C12'—C13'—H13E	109.5
C6—C5—C2	122.36 (15)	H13D—C13'—H13E	109.5
C7—C6—C5	121.26 (18)	C12'—C13'—H13F	109.5
C7—C6—Cl1	117.98 (15)	H13D—C13'—H13F	109.5
C5—C6—Cl1	120.75 (14)	H13E—C13'—H13F	109.5
C8—C7—C6	119.36 (18)	F3—C14—F2	107.78 (13)
C8—C7—H7	120.3	F3—C14—F1	106.64 (14)
C6—C7—H7	120.3	F2—C14—F1	106.11 (14)
C9—C8—C7	120.29 (18)	F3—C14—C4	113.45 (14)
C9—C8—H8	119.9	F2—C14—C4	111.87 (14)
C7—C8—H8	119.9	F1—C14—C4	110.60 (13)
C8—C9—C10	119.89 (19)	N3—C15—N4	116.16 (15)
C8—C9—H9	120.1	N3—C15—H15	121.9
C10—C9—H9	120.1	N4—C15—H15	121.9
			
C1—N2—N3—C15	−0.87 (18)	C2—C5—C6—Cl1	−0.2 (2)
C2—N2—N3—C15	−175.36 (15)	C5—C6—C7—C8	−0.9 (3)
C15—N4—C1—N2	−0.70 (18)	Cl1—C6—C7—C8	178.59 (15)
C15—N4—C1—N1	−179.05 (17)	C6—C7—C8—C9	0.3 (3)
N3—N2—C1—N4	1.04 (19)	C7—C8—C9—C10	0.1 (3)
C2—N2—C1—N4	175.24 (15)	C6—C5—C10—C9	−0.5 (3)
N3—N2—C1—N1	179.53 (15)	C2—C5—C10—C9	−178.84 (16)
C2—N2—C1—N1	−6.3 (3)	C8—C9—C10—C5	0.0 (3)
C4—N1—C1—N4	175.37 (16)	C4—C3—C11—O1	163.90 (19)
C4—N1—C1—N2	−2.8 (2)	C2—C3—C11—O1	−12.7 (2)
C1—N2—C2—C5	−112.47 (18)	C4—C3—C11—O2	−12.5 (3)
N3—N2—C2—C5	61.02 (19)	C2—C3—C11—O2	170.95 (18)
C1—N2—C2—C3	11.1 (2)	C4—C3—C11—O2'	−42.3 (6)
N3—N2—C2—C3	−175.44 (14)	C2—C3—C11—O2'	141.1 (6)
N2—C2—C3—C4	−8.1 (2)	O1—C11—O2—C12	2.9 (3)
C5—C2—C3—C4	113.82 (18)	O2'—C11—O2—C12	−103.3 (10)
N2—C2—C3—C11	168.67 (13)	C3—C11—O2—C12	179.19 (18)
C5—C2—C3—C11	−69.36 (18)	C11—O2—C12—C13	96.5 (3)
C11—C3—C4—N1	−175.26 (16)	O1—C11—O2'—C12'	−35.7 (14)
C2—C3—C4—N1	1.0 (3)	O2—C11—O2'—C12'	58.5 (11)
C11—C3—C4—C14	0.8 (3)	C3—C11—O2'—C12'	172.2 (10)
C2—C3—C4—C14	177.07 (15)	C11—O2'—C12'—C13'	174.9 (12)
C1—N1—C4—C3	5.2 (2)	C3—C4—C14—F3	66.0 (2)
C1—N1—C4—C14	−171.36 (14)	N1—C4—C14—F3	−117.51 (15)
N2—C2—C5—C10	77.54 (19)	C3—C4—C14—F2	−56.2 (2)
C3—C2—C5—C10	−42.9 (2)	N1—C4—C14—F2	120.30 (15)
N2—C2—C5—C6	−100.70 (18)	C3—C4—C14—F1	−174.22 (16)
C3—C2—C5—C6	138.88 (17)	N1—C4—C14—F1	2.25 (19)
C10—C5—C6—C7	0.9 (3)	N2—N3—C15—N4	0.5 (2)
C2—C5—C6—C7	179.21 (16)	C1—N4—C15—N3	0.1 (2)
C10—C5—C6—Cl1	−178.49 (13)		

### Hydrogen-bond geometry (Å, °)

**Table:**

*D*—H···*A*	*D*—H	H···*A*	*D*···*A*	*D*—H···*A*
N1—H1···N4^i^	0.90 (2)	1.96 (2)	2.843 (2)	166.3 (19)

Symmetry codes: (i) −*x*, −*y*+1, −*z*.
